# Lipopolysaccharide-Activated Canine Platelets Upregulate High Mobility Group Box-1 via Toll-Like Receptor 4

**DOI:** 10.3389/fvets.2021.674678

**Published:** 2021-06-21

**Authors:** Ronald H. L. Li, Caelin Hommel, Nghi Nguyen

**Affiliations:** Department of Surgical and Radiological Sciences, School of Veterinary Medicine, University of California, Davis, Davis, CA, United States

**Keywords:** sepsis, thrombosis, inflammation, *E. coli*, translational medicine

## Abstract

High mobility group box-1 (HMGB1) and the toll-like receptor 4 (TLR4) axis is a key mediator of inflammation. Platelet-derived high mobility group box-1 (HMGB1) may also play a critical role in sepsis-mediated thrombosis resulting in complications like disseminated intravascular coagulation and multiple organ failure. While elevated levels of HMGB1 have been documented in humans and dogs with systemic inflammatory response syndrome and sepsis, a better understanding of how platelet agonists and lipopolysaccharide (LPS) mediate platelet HMGB1 expression would open doors to novel therapies for sepsis-mediated thrombosis. Herein, we sought to determine if canine platelets express HMGB1 in the presence or absence of LPS and agonists (ADP or thrombin) and if surface expression of HMGB1 is dependent on platelet TLR4. Canine platelets were unstimulated (resting) or activated with thrombin or adenosine diphosphate (ADP) in the presence or absence of *Escherichia coli* LPS prior to flow cytometric and western blot analyses for HMGB1 expression. We also treated canine platelets with or without TLR4 function blocking antibody or its isotype control. We discovered that while thrombin upregulated both surface and cellular HMGB1 expression, LPS-mediated activation in the presence of ADP priming led to upregulation of surface HMGB1 expression. This expression was found to be most prominent in platelets that had undergone alpha-granule secretion. Inhibition of TLR4 attenuated LPS-induced HMGB1 expression indicating that exteriorization of HMGB1 may be dependent on the non-genomic pathway of platelet TLR4. Our findings indicate that upregulation of platelet-derived HMGB1 occurs as a result of thrombin or TLR4-mediated activation in dogs. Future studies should explore the translational implication of platelet-derived HMGB1 as novel therapeutic targets in humans and dogs with sepsis.

## Introduction

Sepsis, a life-threatening syndrome caused by dysregulation of the immune system due to microbial infection, is associated with high morbidity and mortality in humans and dogs (1, 2). In humans, the overwhelming host inflammatory response leads to dysregulation and derangements of the coagulation system resulting in life-threatening complications like thrombosis, disseminated intravascular coagulation and multiple organ failure. Dogs with sepsis also experience similar complications due to microvascular thrombosis, tissue ischemia and overproduction of thrombin (3–5). Recent evidence suggests that extracellular proteins, which are released from activated or necrotic cells during systemic infection, may further propagate inflammation and coagulation.

High mobility group box 1 (HMGB1) normally resides within the nucleus of all cell types and is a non-histone protein that binds to DNA and regulates gene expression. As a danger-associated molecular pattern (DAMP), HMGB1 is either passively released from necrotic cells or actively secreted/externalized in response to inflammatory signals. Extracellular HMGB1, once bound to TLR4 or receptor for advanced glycation end products (RAGE) on innate immune cells, elicits a proinflammatory response via cytokine production, chemotaxis, cell proliferation and differentiation (6–8). There is increasing evidence in human medicine that HMGB1 functions as a critical mediator in linking inflammation and thrombosis via toll-like receptor 4 (TLR4) (9). Elevated levels of circulating HMGB1 have been reported in humans with cardiovascular diseases like ischemic stroke, deep vein thrombosis, peripheral arterial disease as well as sepsis and disseminated intravascular coagulation (10–12).

In addition to being the primary effector cell in hemostasis, platelets in multiple species are recognized innate immune cells (13, 14). We previously demonstrated that canine platelets express functional TLR4, which augments platelet activation via the thromboxane A2 pathway (15). Although mature platelets lack nuclei, human and murine platelets have been shown to express cytoplasmic HMGB1, which is upregulated on the membrane surface or secreted following platelet activation (16–18). HMGB1 knockout models in mice also proved that platelet-derived HMGB1 is a key player in mediating thrombosis during sepsis (19). Activated platelets also induce the formation of neutrophil extracellular traps, which are web-like scaffolds of cell-free DNA and granular proteins with prothrombotic properties, by presenting HMGB1 to nearby neutrophils (17). These studies demonstrate that platelets bridge the gap between innate immunity and coagulation via HMGB1 expression. However, little is known if direct activation of platelets by the Gram-negative bacterial cell wall component, LPS, could induce HMGB1 expression.

Increased levels of HMGB1 in circulation have also been documented in dogs with systemic inflammatory response syndrome and gastric dilation-volvulus (20, 21). Although canine platelets have previously been shown to express functional TLR4, no studies, to date, have demonstrated the expression or release of platelet-derived HMGB1 in dogs. Because platelet expression of HMGB1 may account for the interplay between systemic infection and coagulation, a better understanding of the underlying mechanisms of platelet HMGB1 expression in dogs would open doors for translational research in developing novel therapies for preventing and treating sepsis-mediated thrombosis. Herein, we aimed to characterize the expression of HMGB1 in platelets in the presence or absence of LPS and agonists. Furthermore, we aimed to determine if surface expression of HMGB1 on platelets is dependent on activation of platelet TLR4. We hypothesized that platelet activation in response to endotoxins and agonists would upregulate HMGB1 expression, which, in turn, could be modulated by inhibiting platelet TLR4.

## Materials and Methods

### Population

A total of 24 clinically healthy staff- and student-owned dogs were enrolled and randomized across three groups of eight dogs for each experiment. Each dog underwent a physical examination and a complete blood count (HM5, Abaxis, Union City, CA) prior to enrollment. Dogs were not included if they were <5 kg, on any medications, vaccinated within 30 days prior to participation or had any abnormalities on hematological examination. For the purpose of this study, dogs <6 years of age were categorized as Age Group 1 while dogs >6 years of age were categorized as Age Group 2. This animal study was reviewed and approved by the Institutional Animal Care and Use Committee at the University of California, Davis (#20496) and written, informed consent was obtained for all animals.

### Preparation of Gel-Filtered Platelets

Approximately 4–6 ml of blood was collected from the jugular or cephalic vein and immediately transferred to blood tubes containing 3.2% sodium citrate. Following gentle inversion, blood tubes were visually inspected and confirmed to be free of clots. A complete blood count was obtained using an automated analyzer (HM5, Abaxis, Union City, CA). After transferring to round-bottom polypropylene tubes, whole blood was placed in 37°C bead bath for 30 min before centrifugation at 300 × *g* (5 min, room temperature, no brakes). Platelet rich plasma was then filtered through a Sepharose 2B column and eluted with Tyrodes HEPES buffer (pH 7.2, 5 mM dextrose without divalent cations) at 37°C. A platelet count of gel-filtered platelets was obtained by an automated analyzer (HM5, Abaxis, Union City, CA) and confirmed by blood smear evaluation. Platelets were further diluted to a final concentration of 1 × 10^7^ cells/ml for flow cytometry experiments and 1 × 10^8^ cells/ml with Tyrodes HEPES buffer (pH 7.2, 5 mM dextrose, 0.5% heat-inactivated canine serum) for SDS-PAGE and western blot analysis.

### Platelet Activation

Gel-filtered platelets were stimulated with 5 μg/mL LPS (*Escherichia coli* 0111:B4, EMD Millipore, Temecula, CA) (30 min, 37°C), 10 μM ADP (MilliporeSigma, St. Louis, MO) or 0.01 U/mL bovine α-thrombin (Haematologic Tecnologies, Essex Junction, VT) for 15 min at 37°C. Additionally, platelets were treated with 5 μg/ml LPS for 30 min after priming with ADP (15 min at 37°C).

### Inhibition of Toll-Like Receptor 4

Platelet TLR4 was inhibited as previously described (15). Briefly, washed platelets (1 × 10^7^ cells/ml) were treated with a function blocking monoclonal mouse anti-human TLR4 (CD284) antibody (50 μg/ml, low endotoxin, clone HTA125, BioRad, Hercules, CA) for 30 min at room temperature prior to activation. The same concentration of mouse IgG2a isotype (low endotoxin, clone OX-34, BioRad, Hercules, CA) was used as an isotype control.

### Detection of Surface HMGB1 and P-selectin by Flow Cytometry

All samples were incubated with monoclonal mouse anti-human HMGB1 antibody conjugated to Alexa Fluor 488 (1:20, Clone:115603, R&D Systems, Minneapolis, MN). The amino acid sequence of human HMGB1 is shown to have 100% alignment with canine HMGB1 (FASTA Query: 33771, BLAST, NIH). Platelet integrin β_3_ was detected by mouse anti-human monoclonal antibodies to CD61 conjugated to allophycocyanin (1:1,000, clone: VI-PL2, eBioscience, Carlsbed, CA). Platelet P-selectin was detected by biotinylated monoclonal antibodies to CD62P (1:100, clone: RB40.34, BD Pharmingen, San Jose, CA) for 45 min at 37°C, followed by incubation with streptavidin conjugated to phycoerythrin-Cy7 (1:100, Invitrogen, Carlsbed, CA) for 30 min at 37°C. Samples were then fixed in 1% paraformaldehyde for 30 min in room temperature and stored at 4°C for further analysis.

Flow cytometry was performed to detect surface HMGB1 and P-selectin. Compensation beads and monoclonal immunoglobulin G1 kappa conjugated to matched experimental fluorochromes were used to calculate compensation matrixes for correction and removal of spillover fluorescence. Platelets were identified by forward scatter and side scatter properties and by 0.9 and 3 um calibration beads as previously described (Supplementary Figure 1A). Gating boundaries were used by fluorescence-minus-one and isotype controls (Supplementary Figures 1B,C). HMGB1 and P-selectin expression were measured as percentage of positive platelets or median fluorescence intensity (MFI). The extent of HMGB1 upregulation was measured as fold change (FC) in MFI on a log_10_ scale between activated and resting platelets calculated using the following formula:

$$\begin{array}{ll} \text{lo}{\text{g}}_{10}\text{ fold change in MFI }= & \left(\text{Lo}{\text{g}}_{10}\text{ MF}{\text{I}}_{\text{Activated}}\right)\\ - & \left(\text{Lo}{\text{g}}_{10}\text{ MF}{\text{I}}_{\text{Resting}}\right) \end{array}$$

### Detection of Platelet HMGB1 Expression by Western Blot Analysis

Platelets (1 × 10^8^/ml) were separated out by centrifugation at 5,000 × *g* for 1 min at room temperature. Pellets were resuspended in EDTA buffer containing protease inhibitor (Halt Protease Inhibitor, Thermo Scientific, Rockford, IL) before addition of 1x Laemmli containing 355 mM of reducing agent 2-mercaptoethanol (BME) (Bio-Rad, Hercules, CA). Samples were then boiled at 100°C for 5 min, placed on ice for 5 min and stored at−20°C until analysis within 6 months. Proteins were separated by sodium dodecyl sulfate–polyacrylamide gel electrophoresis (SDS-PAGE), transferred to polyvinylidene fluoride membranes and stained with 0.1% Ponceau S to confirm equal and adequate protein transfer. After blocking in 10% bovine serum albumin (BSA), and washed 5 times in TBST, membranes were incubated in monoclonal mouse anti-HMGB1 antibodies (1:2,000 diluted in 5% BSA, clone:115603, R&D Systems, Minneapolis, MN) for 2 h at room temperature. After washing, membranes were probed with a polyclonal donkey anti-mouse antibody conjugated to horseradish peroxidase (1:2,000 diluted in 5% BSA, clone:115603, R&D, Minneapolis, MN) for 2 h at room temperature. Membranes were exposed to a chemiluminescent substrate (WesternBright Quantum, Advansta, Menlo Park CA), and imaged with a commercial imaging system (FluorChem E, ProteinSimple). Annotation was added for labeling of the molecular marker on the imaging system. The expected migration of proteins based on the predicted molecular mass of HMGB1 and beta-actin was 25–29 and 47 kDa, respectively. A notable 2 molecular weight species of HMGB1 was noted in all replicates of blots. Immunoblotting and imaging for HMGB1 were performed first followed by stripping of primary and secondary antibodies using commercially available stripping buffer (Restore PLUS Western Blot Stripping Buffer, Thermo Scientific) for 15 min at room temperature. Actin, served as loading control, was detected by a monoclonal mouse anti-beta actin antibody (1:2,000, ab8224, Abcam, Cambridge, MA), followed by goat anti-mouse secondary antibody conjugated to horseradish peroxidase (1:20,000, ab97040, Abcam, Cambridge, MA). Densitometry was quantified using available software (ImageJ, NIH) as previously described (22), and total cellular expression of HMGB1 was expressed as relative density to the respective loading control in each lane using β-actin (HMGB1:Actin).

### Detection of Platelet HMGB1 Secretion via ELISA

Platelets (1 × 10^8^/ml) were separated out by centrifugation at 5,000 × *g* for 1 min at room temperature. Supernatant was collected with the addition of protease inhibitor (1x HALT, Thermofisher) prior to flash frozen in liquid nitrogen and stored at −80°C before analysis. Platelet supernatant was analyzed undiluted using commercially available HMGB1 ELISA (TECAN, Männedorf, Switzerland), previously validated for use in canine plasma (21).

### Statistical Analysis

Normality of data was assessed visually and by Shapiro-Wilk normality test. Percentage of positive events on flow cytometry underwent arcsine transformation. One-way repeated measures ANOVA or its non-parametric equivalent, Friedman test, was used to assess the effect of platelet activation or TLR4 inhibition on HMGB1 expression, followed by *post-hoc* analysis by Tukey's or Dunn's multiple comparisons testing as appropriate. Paired data were compared using either *t*-tests for normally distributed data or Wilcoxon signed-rank test for non-parametric data. Data that violated the assumption of sphericity were analyzed using Greenhouse-Geisser correction. Normally distributed data were presented as mean ± standard deviation while non-parametic data were presented as median and interquartile range (IQR). A *p*-value < 0.05 was considered statistically significant. Data were analyzed using commercially available software (Prism 7.0, GraphPad Software, La Jolla, CA).

## Results

A total of 24 dogs were studied. The median age of dogs was 5.0 years (range: 1.0–14.0). Of the 24 dogs, 7 were female spayed and 18 were male neutered. Nineteen dogs were mixed breed and 5 were pure breeds including 1 German Sheppard, 2 Border Collie, 1 Golden Retriever, and 1 was Brittany Spaniel.

### Thrombin Upregulates HMGB1 Expression and Causes Secretion of HMGB1 in Canine Platelets

In general, HMGB1 expression on the surface of resting platelets was highly variable among dogs (29.03% ± 11.38) with a coefficient of variation of 39.22%.

**Figure 1:**
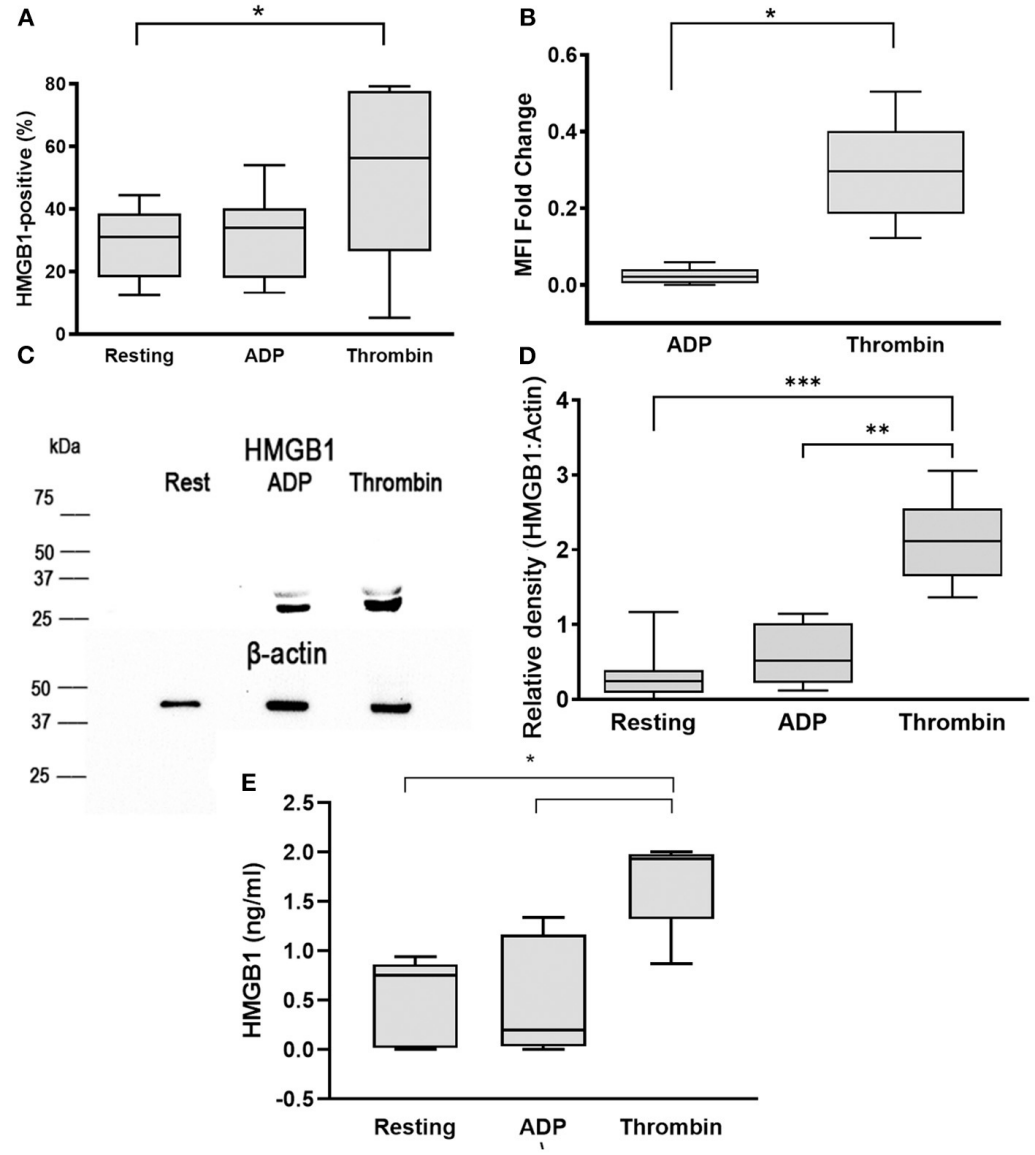
Thrombin upregulates surface and cellular HMGB1 expression in platelets. Canine washed platelets from 8 dogs were either unstimulated (resting) or activated with 10 μM ADP or 0.01 U/ml thrombin. Surface HMGB1 expression was measured by flow cytometry and expressed as percent (%) of HMGB1-positive platelets or median fluorescence intensity (MFI) fold change on a log_10_ scale. **(A,B)** Thrombin-activated platelets significantly upregulated surface HMGB1 expression compared to resting or ADP-treated platelets. **(C)** Representative immunoblot of HMGB1 (~25–29 kDa) and β-actin (~42 kDa) (loading control) in platelet lysate after stimulation with 10 μM ADP or 0.01 U/ml thrombin **(D)**. Total cellular HMGB1 expression in 1 ×10^8^ platelets/ml was assessed by densitometry of western blots relative to β -actin, used as loading control in resting, ADP- and thrombin-activated platelets in 6 dogs with 2 replicates in each dog with identical results. Thrombin significantly upregulated cellular HMGB1 expression compared to resting and ADP-activated platelets. Regions of the immunoblot were cropped for the individual figure. **(E)** Platelet supernatant was further analyzed for soluble HMGB1 via ELISA. Each box represents the 25th and 75th quartiles and the line represents the median. Whiskers represent the range of data. *p < 0.05, **p < 0.001, ***p < 0.0005. Pairwise comparison between ADP- and thrombin-activated platelets was conducted using paired *t*-test. One-way repeated measures ANOVA was used to assess the effect of platelet activation on HMGB1 expression, followed by *post-hoc* analysis by Dunnett's multiple comparison.

To evaluate HMGB1 expression on the platelet surface, washed platelets were unstimulated (resting) or activated with 10 μM ADP or 0.01 U/ml thrombin prior to flow cytometry analysis. We found that ADP did not have a significant impact on surface HMGB1 expression (31.73% ± 13.54, *p* = 0.053). However, thrombin, one of the most potent platelet agonists in dogs, resulted in significant elevation in HMGB1 surface expression (50.45% ± 27.76) compared to resting platelets (*p* = 0.036) (Figure 1A). The extent of surface HMGB1 upregulation, measured as MFI FC on a log_10_ scale from resting platelets, was also significantly higher in thrombin-treated platelets compared to ADP-treated platelets (0.30 ± 0.13 vs. 0.024 ± 0.020, *p* = 0.0017) (Figure 1B).

To further evaluate the effect of platelet agonists on total cellular expression of HMGB1, we semi-quantitatively measured HMGB1 by Western blot and densitometry analyses in resting, ADP, and thrombin-activated platelets (Figure 1C). Total cellular expression of HMGB1 was notably higher in thrombin-treated platelets (HMGB1:Actin = 2.34, IQR: 1.82–2.71) compared to ADP (HMGB1:Actin = 0.44, IQR: 0.16–0.68, *p* = 0.03) and resting platelets (HMGB1:Actin = 0.28, IQR: 0.12–0.59, *p* = 0.031) (Figures 1C,D).

We next assessed if the upregulation of HMGB1 is associated with increased secretion of HMGB1 by analyzing soluble HMGB1 using ELISA. We found that thrombin activation not only upregulated surface expression of HMGB1, but also caused greater secretion of HMGB1 (1.86 ng/ml, IQR: 1.10–1.96) compared to resting platelets (0.77 ng/ml, IQR: 0.19–0.90, *p* = 0.03) and ADP-stimulated platelets (0.53 ng/ml, IQR: 0.015–1.25, *p* = 0.01) (Figure 1E).

### LPS in the Presence of ADP Upregulates Surface HMGB1 Expression but Not Cellular Expression

**Figure 2:**
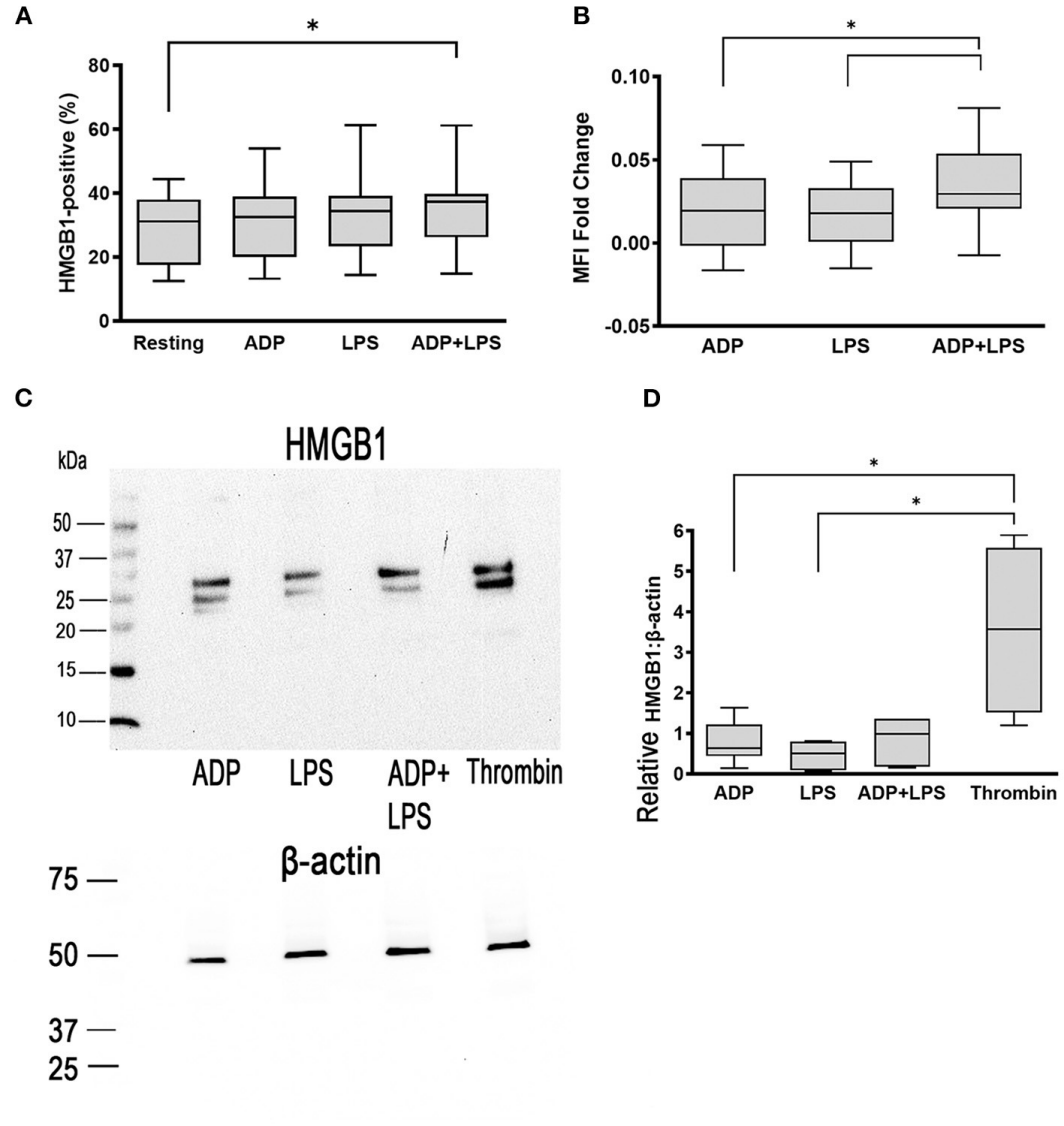
ADP priming in the presence of LPS upregulates surface HMGB1 expression. Washed platelets from 8 dogs were unstimulated (resting) or activated with 5 μg/ml LPS or 10 μM ADP. Platelets were also primed with 10 μM ADP prior to treatment with LPS. **(A,B)** Surface HMGB1 expression from 8 dogs was assessed by flow cytometry and measured as percent (%) positive or median fluorescence intensity (MFI) fold change on a log_10_ scale. While LPS alone did not augment surface HMGB1 expression, LPS in the presence of ADP upregulated HMGB1 expression. **(C,D)** Total cellular HMGB1 expression in 1 ×10^8^ platelets/ml was further assessed by western blot and relative densitometry calculated based on beta-actin, used as loading control in 6 dogs with 2 replicates in each dog with identical results. **(C)** Representative immunoblot of HMGB1 (~25–29 kDa) and β-actin (~42 kDa) in platelet lysate after stimulation with 10 μM ADP, 5 μg/ml LPS or LPS in the presence of ADP. Thrombin (0.01 U/ml) served as positive control. LPS alone or LPS in the presence of ADP did not upregulate total cellular HMGB1 expression. Each box represented the 25th and 75th quartiles and line represents the median. Whiskers represent the range of data. *p > 0.05. One-way repeated measures ANOVA was used to assess the effect of platelet activation on HMGB1 expression, followed by *post-hoc* analysis by Tukey multiple comparisons.

To determine if LPS could stimulate canine platelets to upregulate HMGB1, we treated washed canine platelets with 5 μg/ml LPS and measured surface and cellular HMGB1 expression by flow cytometry and western blot analysis. We previously showed that LPS has a limited capacity to activate canine platelets and, as expected, LPS treatment did not result in a significant elevation in HMGB1-positive platelets (33.11% ± 13.51) compared to resting (*p* = 0.13) or ADP-treated platelets (*p* = 0.13) (Figure 2A). Like ADP (0.019 ± 0.024, *p* = 0.98), LPS also had minimal impact on HMGB1 MFI fold change on a log_10_ scale (0.017 ± 0.020) (Figure 2B).

LPS treatment after priming with 10 μM ADP significantly increased surface HMGB1 expression when compared to resting platelets (35.07% ± 12.86 vs. 29.42% ± 11.01, *p* = 0.017) (Figure 2A). ADP priming prior to LPS treatment of platelets also resulted in upregulation in HMGB1 (0.034 ± 0.026) compared to platelets treated with either ADP (0.019 ± 0.024, *p* = 0.036) or LPS (0.017 ± 0.02) alone (*p* = 0.020) (Figure 2B). However, we did not find significant upregulation in cellular HMGB1 expression in platelets treated with LPS and ADP (HMGB1:Actin = 0.81 ± 0.56) compared to platelets treated with ADP (HMGB1:Actin =0.81 ± 0.53, *p* = 0.51)or LPS alone (HMGB1:Actin = 0.42 ± 0.32, *p* > 0.99) by western blot analysis. Thrombin remained the most potent agonist in inducing HMGB1 expression (Figures 2C,D).

### LPS-Mediated Surface HMGB1 Expression Is Upregulated in Activated Platelets

**Figure 3:**
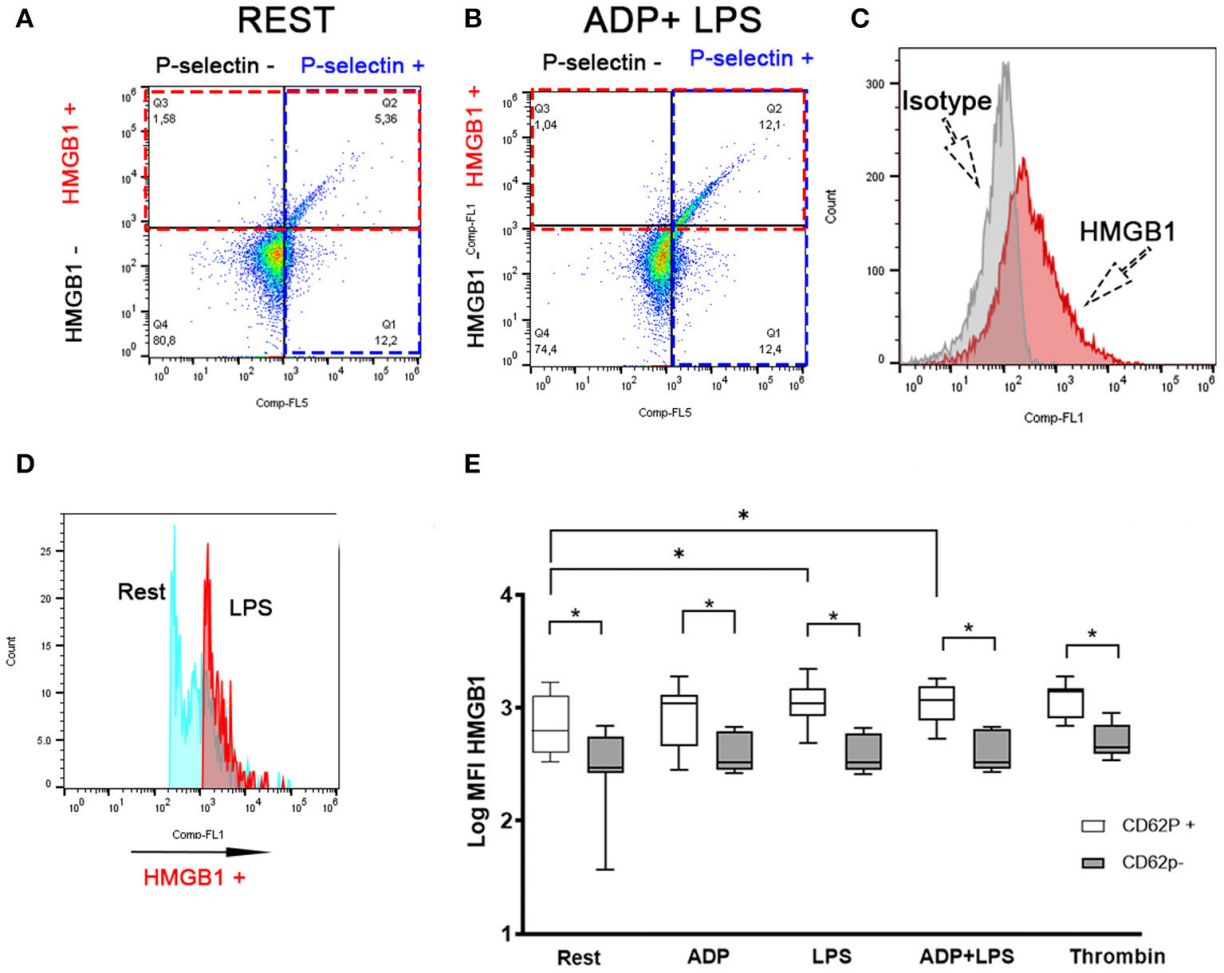
LPS-mediated HMGB1 upregulation occurs in activated platelets. Representative scatter dot plots in unstimulated (rest) **(A)** and platelets treated with 10 μM ADP and 5 μg/ml LPS **(B)** illustrating that canine platelets express HMGB1 mostly in P-selectin positive (intersection between red and blue box) platelets. **(B)** LPS in the presence of ADP leads to an increased number of cells co-expressing HMGB1 and P-selectin (Intersection between red and blue boxes). **(C)** Representative histograms demonstrating isotype control and HMGB1-positive platelets. **(D)** Representative histograms demonstrating the upregulation of surface HMGB1 between unstimulated resting (blue) and LPS-treated (pink) platelets. **(E)** Box and whisker box plots of surface HMGB1 expression, measured as median fluorescence intensity (MFI) (on a log_10_ scale) by flow cytometry in eight dogs. Activated platelets shown here as P-selectin (CD62P) positive platelets expressed significantly more HMGB1 than CD62P negative platelets. Only CD62P positive platelets showed an increase response to LPS in the absence or presence of ADP. Each line represents the median while the box represents the 25th and 75th percentile. Whiskers represent the range. Paired *t*-test was used to compare HMGB1 expression between CD62P positive and negative platelets in each treatment while one-way ANOVA of repeated measures was used to assess treatment-effect on HMGB1 expression for either CD62P positive or CD62P negative platelets. This is then followed by *post-hoc* analyses by Tukey's test. *p < 0.05.

To evaluate the association between surface HMGB1 expression and platelet activation, we labeled canine platelets for surface P-selectin, a marker of α-granule secretion, as well as HMGB1. Isotype control of HMGB1 expression is shown in Figure 3C. Surface expression of HMGB1 occurs almost exclusively on activated platelets based on co-expression of P-selectin and HMGB1 (Figures 3A,B). We found that platelets that underwent α-granule secretion (P-selectin positive) had significantly higher surface HMGB1 expression than those that did not, regardless of treatments (*p* ≤ 0.02) (Figure 3E). Interestingly, only P-selectin-positive platelets showed significant upregulation of surface HMGB1 in response to LPS (Log_10_ MFI: 3.03 ± 0.16) compared to resting platelets (Log_10_ MFI: 2.75 ± 0.26, *p* = 0.036) (Figures 3D,E). This observation was consistent in ADP-primed platelets treated with LPS (Figures 3D,E). Thrombin-mediated upregulation of surface HMGB1 (Log_10_ MFI: 2.70 ± 0.15) was found only in P-selectin negative platelets when compared to ADP (2.60 ± 0.17, *p* = 0.0015), LPS (2.59 ± 0.17, *p* = 0.0030) or ADP in the presence of LPS (2.60 ± 0.17, *p* = 0.0026) (Figure 3E).

LPS (0.68 ng/ml; IQR: 0–1.15) and LPS treatment in the presence of ADP (0.91 ng/ml; IQR: 0–2.22) did not result in significant elevation in the release of soluble HMGB1 compared to resting platelets (0.75 ng/ml; IQR 0.015–0.87, *p* > 0.1).

### LPS-Mediated Upregulation of HMGB1 Surface Expression Is Dependent on TLR4

**Figure 4:**
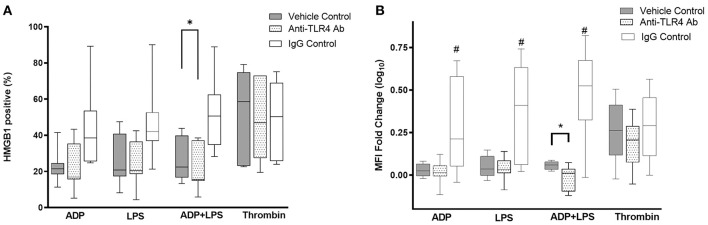
LPS-mediated HMGB1 expression is dependent on platelet TLR4. Washed platelets from eight dogs were treated with function blocking antibodies, an isotype control or vehicle control prior to activation with 10 μM ADP, 5 μg/ml LPS, 0.01 U/ml thrombin or LPS in the presence of ADP. HMGB1 surface expression, measured by flow cytometry, was expressed as **(A)** percent (%) positive cells or **(B)** median fluorescence intensity (MFI) fold change on a log_10_ scale. TLR4 inhibition modulated LPS-mediated HMGB1 expression in platelets primed with ADP. Each line represents the median while the box represents the 25th and 75th percentile. Whiskers represent the range. One-way repeated measures ANOVA was used to assess the effect of TLR4 inhibition on HMGB1 expression separately in ADP, ADP+LPS or thrombin activated platelets, followed by *post-hoc* analysis by Dunnett's multiple comparisons. *p < 0.05, ^#^*p* < 0.01.

Canine platelets have previously been shown to express functional TLR4. To determine if LPS-mediated HMGB1 surface expression were dependent on TLR4, canine washed platelets were first treated with a TLR4 function blocking antibody or its isotype control before treating platelets with LPS in the presence or absence of ADP. To ensure that platelets were neither inhibited nor activated by the TLR4 antibodies, platelets were also activated by ADP. As expected, TLR4 inhibition resulted in a significant reduction in HMGB1 positive platelets in those treated with LPS in the presence of ADP (20.59% ± 12.33 vs. 26.74% ± 11.66, *p* = 0.010) (Figure 4A). Inhibition of TLR4 also modulated the extent of HMGB1 upregulation (MFI FC log_10_ scale: −0.018 ± 0.074 vs. 0.057 ± 0.023, *p* = 0.040) in response to ADP and LPS (Figure 4B). Inhibition of TLR4, however, had no impact on HMGB1 expression in platelets treated with ADP (MFI FC log_10_ scale: 0.017 ± 0.068 vs. 0.028 ± 0.037, *p* = 0.65; 22.25% ± 12.98 vs. 22.97 ± 9.26, *p* = 0.86) or LPS alone (MFI FC log_10_ scale: 0.035 ± 0.066 vs. 0.050 ± 0.063, *p* = 0.53; 25.86% ± 13.76 vs. 23.31% ± 12.61, *p* = 0.063). Interestingly, isotype controls upregulated HMGB1 in platelets treated with ADP, LPS or the combination of both.

Since HMGB1 has previously been shown to mediate platelet activation via platelet TLR4, we inhibited TLR4 in thrombin-activated platelets to examine if surface HMGB1 can be upregulated by platelet-derived HMGB1. When TLR4 function blocking antibody was added to platelets prior to being activated by 0.01 U/mL thrombin, there was a trend in HMGB1 downregulation (MFI FC log_10_ scale: 0.19 ± 0.15 vs. 0.26 ± 0.18) but this difference did not reach statistical significance (*p* = 0.21).

## Discussion

We demonstrated that canine platelets express HMGB1 and its cellular expression is upregulated in response to thrombin. We also showed that surface HMGB1 expression in platelets is upregulated by endotoxin or LPS via platelet TLR4. Based on the results of this study, HMGB1 is strongly expressed in thrombin-activated platelets. Further studies are needed to ascertain if HMGB1 may act as a danger-associated molecular pattern to further propagate platelet activation in a positive feedback loop.

HMGB1, a nuclear binding protein, once exteriorized, serves as a mediator of inflammation and coagulation, and hence represents as a novel treatment target in sepsis and thrombosis. While our findings are consistent with the current knowledge that thrombin-mediated activation in human platelets leads to HMGB1 externalization from the cytoplasm, we also found that thrombin upregulates cellular expression of HMGB1 (23). Similar to proteins like plasminogen activator inhibitor 1 and Bcl-1, which undergo rapid *de novo* synthesis in response to thrombin, the rapid synthesis of HMGB1 suggests that thrombin-mediated signaling results in a surge of translation of the constitutively present HMGB1 mRNA, previously found in the human platelet transcriptome (23–25). The 2 molecular weight species of HMGB1 noted in our Western blots also suggest that newly synthesized platelet HMGB1 may reside within the cytosol and plasma membrane. The lower molecular weight species are suggestive of nascent and newly synthesized protein, whereas the higher molecular weight species represent a more mature, post-translationally modified isoform in the plasma membrane (26). There are 2 important implications to our findings: (1) HMGB1 synthesis and translation of HMGB1 mRNA in platelets is signal-dependent. Considering that platelet-derived HMGB1 augments thrombosis by activating nearby neutrophils and platelets, we speculate that the expression of HMGB1 is highly controlled, or silenced in platelets under resting conditions. (2) Consumption of anticoagulant factors and subsequent elevation in thrombin generation due to sepsis and other systemic inflammatory disease could lead to upregulation in platelet-derived HMGB1. In cultured macrophages, HMGB1 synthesis and mRNA translation are dependent on protein kinase C, and PI-3/AKT pathways, both of which are activated by thrombin-mediated signaling in platelets (27). Our findings suggest that increased thrombin generation in sepsis or other inflammatory diseases associated with hypercoagulability could lead to overzealous production of platelet-derived HMGB1, previously shown to be associated with morbidity and mortality in human septic patients. Post transcriptional control of HMGB1 mRNAs in platelets will be an important future direction as this knowledge will offer the possibility to target transcriptional pathways with novel therapies.

Lipopolysaccharide from the Gram-negative bacterial cell wall has been demonstrated to induce inflammation by promoting pro-inflammatory cytokines and release of HMGB1 in innate immune cells. In this study we revealed an additional role that platelets play in innate immunity. We demonstrated that engagement of TLR4 by *E. coli* LPS has a limited capacity in triggering upregulation of surface HMGB1. Because total cellular expression of HMGB1 in canine platelets is not impacted by LPS treatment, TLR4 activation may likely result in translocation of HMGB1 from the cytoplasm to the cell surface. Similar to a study by Rivadeneyra et al. our previous study demonstrated that TLR4 activation in canine platelets only results in modest alpha-granule secretion. With ADP priming prior to LPS stimulation, we previously found increased activation of the cyclooxygenase pathway causing increased thromboxane A2 secretion in canine platelets (15). Nocella et al. proposed that activation of TLR4 and platelet ADP receptors causes amplification of the Akt and p38 MAP kinase axis leading to phospholipase A2 phosphorylation and generation of thromboxane A_2_. Blocking of cyclooxygenase by aspirin in our previous study mitigated TLR4 signaling resulting in decreased alpha-granule release (15). Although the sensing pathways of LPS and ADP leading to surface upregulation of platelet HMGB1 is still unknown, there is evidence that activation of phosphoinositol 3 kinase and Akt in cardiomyocytes stimulates HMGB1 synthesis (28, 29). Alternatively, longer incubation time with LPS and ADP may be required to elicit *de novo* synthesis of HMGB1 in canine platelets.

Platelet-derived HMGB1 has been shown to not only mediate inflammation, and bacterial clearance, it also facilitates a prothrombotic cascade (11). HMGB1, once exteriorized or released in the platelet milieu, can, in theory, activate platelets to further release HMGB1 in an autocrine or paracrine fashion via TLR4. However, we did not find that TLR4 inhibition alone suppresses HMGB1 expression. This suggests that canine platelets may also express other receptors like RAGE that respond to HMGB1. A study by Ahrens et al. previously demonstrated that thrombin upregulates surface expression of RAGE in human platelets and that the binding of recombinant HMGB1 on platelets was minimal in RAGE knockout platelets. These findings suggest that RAGE may also be a major receptor for HMGB1 in canine platelets (30). Another explanation is that secreted or bound HMGB1 may activate platelets via TLR2, although its expression in canine platelets has yet to be determined. HMGB1 has been shown to activate monocytes through RAGE and TLR2 to release cytokines and upregulate tissue factor (11). Further investigations in the HMGB1-RAGE-TLR4/TLR2 axis in platelet activation using recombinant HMGB1 or platelet-derived HMGB1 and knockouts will be warranted in dogs considering that RAGE-dependent mechanisms may be central to the pathogenesis of thrombosis in sepsis.

This study has some limitations. First, we did not utilize microscopy to confirm the intracellular location of HMGB1. Super resolution microscopy, electron microscopy, or subcellular Western blot analysis would verify if newly synthesized HMGB1 localizes within the cytoplasm, granules, or plasma membrane of canine platelets. In addition, microscopy would provide more information on whether externalized HMGB1 corresponds to fibrinogen binding and exteriorization of the phospholipid, phosphatidylserine. Second, the mechanism of TLR4-signaling pathway leading to HMGB1 upregulation in canine platelets was not explored in this study. Given that plasma HMGB1, regardless of its sources, is associated with mortality and morbidity in dogs, further characterization of platelet secretion of HMGB1 is needed (20, 31). Further studies including knockout models in mice or blocking of TLR4-signaling pathways would confirm the translational significance of dogs as a naturally occurring model for sepsis. Also, we did not investigate the underlying mechanisms responsible for the upregulation of HMGB1 or characterize the post-transcriptional control of mRNA in activated platelets. Lastly, while all study dogs were deemed to be clinically healthy, the highly variable expression of platelet HMGB1 among the subjects warrants further investigations in a larger population of dogs.

In conclusion, this is the first study to document expression of HMGB1 in canine platelets. The finding of surface expression of HMGB1 in LPS-activated platelets via TLR4 offers a contribution to the study of translational sepsis research. This study suggests that dogs may serve as a viable translational model for the study of sepsis-mediated thrombosis and the future studies of novel therapeutics in sepsis.

## Data Availability Statement

The raw data supporting the conclusions of this article will be made available by the authors, without undue reservation.

## Ethics Statement

The animal study was reviewed and approved by Institutional Animal Care and Use Committee at the University of California, Davis. Written informed consent was obtained from the owners for the participation of their animals in this study.

## Author Contributions

RL designed and supervised the experiments and was actively involved in drafting and editing of the manuscript. CH and NN were responsible for conducting the experiments, data gathering, statistical analysis, and drafting of the manuscript. All authors agree to be accountable for the content of the work.

## Conflict of Interest

The authors declare that the research was conducted in the absence of any commercial or financial relationships that could be construed as a potential conflict of interest.

## Supplementary Material

Supplementary Figure 1 Gating strategies and isotype controls for detecting canine platelets. Representative scatter plot diagram of flow cytometric analysis of gel-filtered platelets from a dog. **(A)** Platelets were unstimulated, or activated with 10 μM ADP, or 0.01 U/ml thrombin. Platelets were identified based on their forward (FS) and side scatter (SS) profile. Note the change in FS and SS as platelets were activated in the presence of ADP or thrombin. **(B)** Fluorescence minus one controls were used to establish gating for P-selectin positive platelets in resting and ADP-activated platelets. **(C)** Unstimulated platelets from another dog stained with an isotype control or mouse anti-human monoclonal antibodies to CD61 (integrin β_3_) conjugated to allophycocyanin. Activated platelets in the presence of 0.01 U/ml thrombin were stained with isotype control or biotinylated monoclonal antibodies to CD62P (P-selectin).
