# Supplementary figures and images for: Lipopolysaccharide-Activated Canine Platelets Upregulate High Mobility Group Box-1 via Toll-Like Receptor 4
Source: Front Vet Sci. 2021 Jun 21;8:674678. doi: 10.3389/fvets.2021.674678 (PMC8255672; doi:10.3389/fvets.2021.674678)

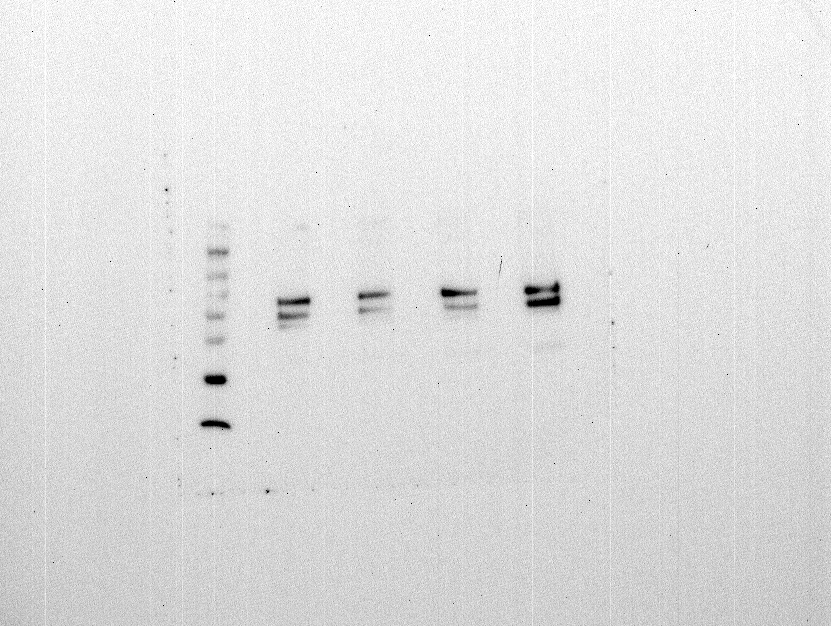

Supplement: Supplementary file 1 [file Data_Sheet_1.zip › 2018-08-30_Reckon Cell R_A_L_AandL 10s-30s-45s-1min_8bit (1) (1).tif]

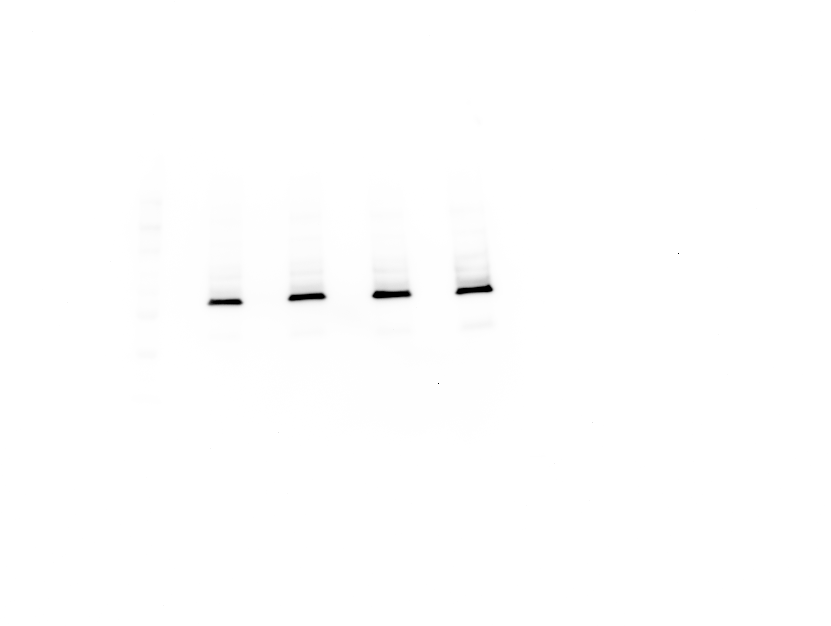

Supplement: Supplementary file 1 [file Data_Sheet_1.zip › 2018-08-31_Reckon Cell Actin R_A_L_AandL 1min_8bit (1).tif]

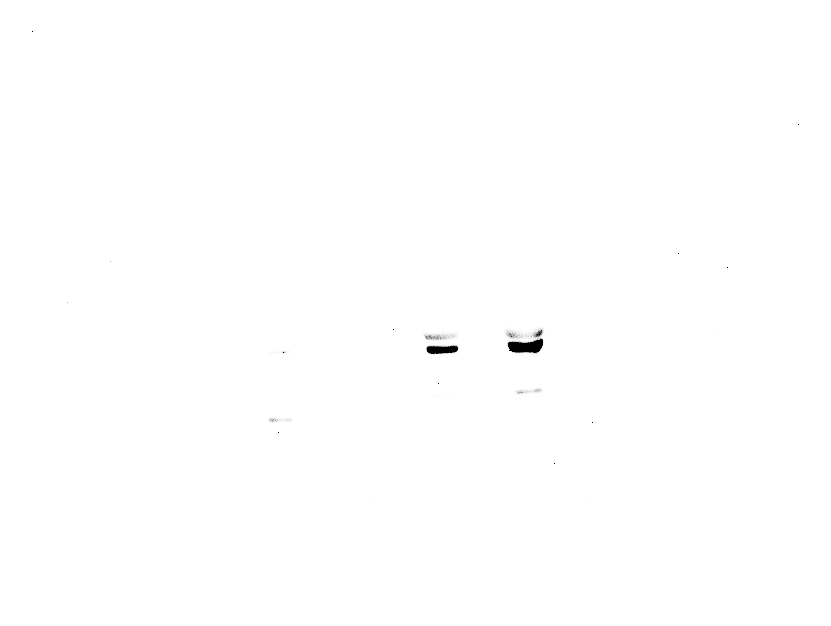

Supplement: Supplementary file 1 [file Data_Sheet_1.zip › 2019-04-05_Lira Cell R-A-T_8bit (1).tif]

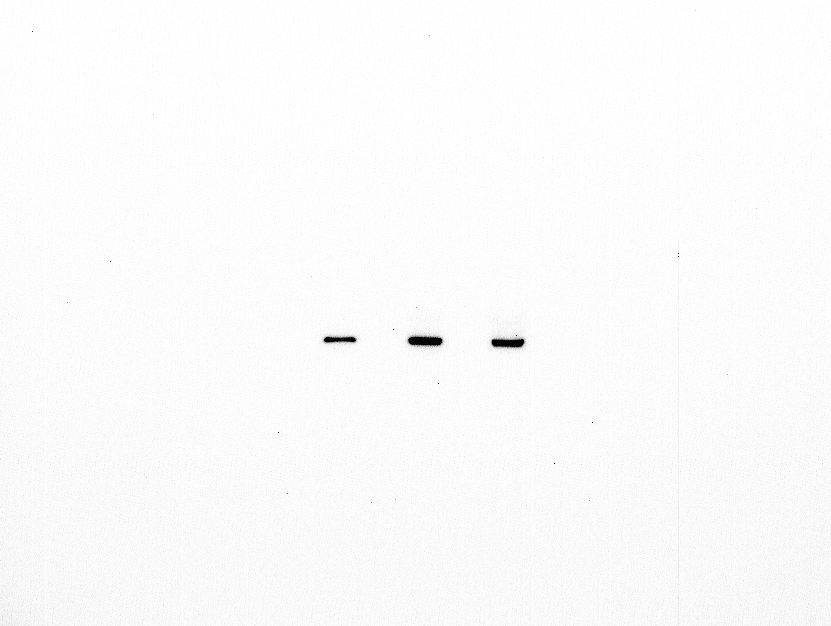

Supplement: Supplementary file 1 [file Data_Sheet_1.zip › 2019-04-09_Lira Cell Actin R-A-T_8bit (1).tif]

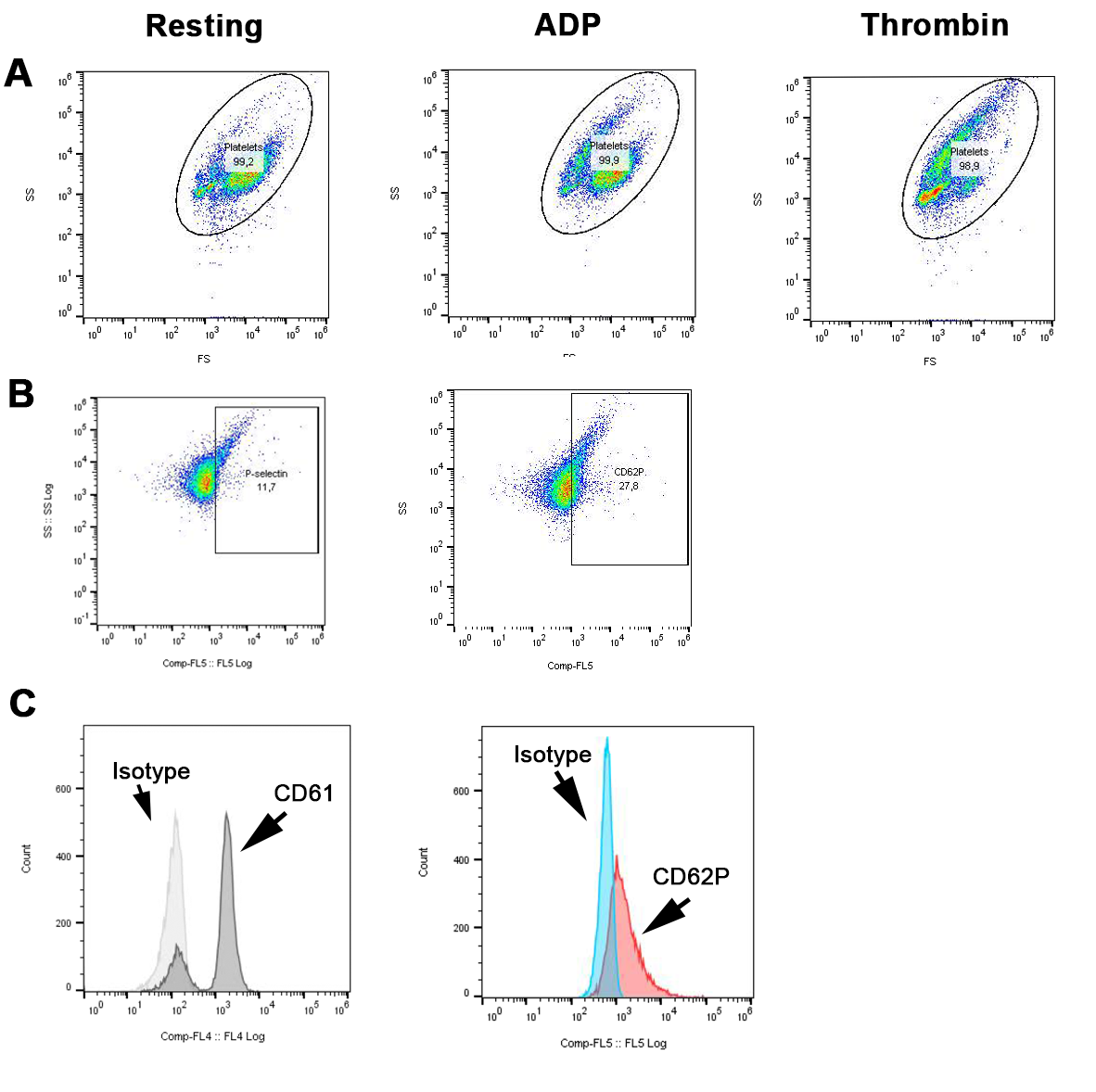

Supplement: Supplementary Figure 1 — Gating strategies and isotype controls for detecting canine platelets. Representative scatter plot diagram of flow cytometric analysis of gel-filtered platelets from a dog. (A) Platelets were unstimulated, or activated with 10 μM ADP, or 0.01 U/ml thrombin. Platelets were identified based on their forward (FS) and side scatter (SS) profile. Note the change in FS and SS as platelets were activated in the presence of ADP or thrombin. (B) Fluorescence minus one controls were used to establish gating for P-selectin positive platelets in resting and ADP-activated platelets. (C) Unstimulated platelets from another dog stained with an isotype control or mouse anti-human monoclonal antibodies to CD61 (integrin β3) conjugated to allophycocyanin. Activated platelets in the presence of 0.01 U/ml thrombin were stained with isotype control or biotinylated monoclonal antibodies to CD62P (P-selectin). [file Image_1.tif]
